# Accuracy and sources of error of out‐of field dose calculations by a commercial treatment planning system for intensity‐modulated radiation therapy treatments

**DOI:** 10.1120/jacmp.v14i2.4139

**Published:** 2013-03-04

**Authors:** Jessie Y. Huang, David S. Followill, Xin A. Wang, Stephen F. Kry

**Affiliations:** ^1^ Graduate School of Biomedical Sciences The University of Texas Health Science Center Houston TX; ^2^ Department of Radiation Physics The University of Texas M. D. Anderson Cancer Center Houston TX USA

**Keywords:** out‐of‐field dose, TPS, dose calculation, accuracy, IMRT

## Abstract

Although treatment planning systems are generally thought to have poor accuracy for out‐of‐field dose calculations, little work has been done to quantify this dose calculation inaccuracy for modern treatment techniques, such as intensity‐ modulated radiation therapy (IMRT), or to understand the sources of this inaccuracy. The aim of this work is to evaluate the accuracy of out‐of‐field dose calculations by a commercial treatment planning system (TPS), ${\text{Pinnacle}}^{3}$ v.9.0, for IMRT treatment plans. Three IMRT plans were delivered to anthropomorphic phantoms, and out‐of‐field doses were measured using thermoluminescent detectors (TLDs). The TLD‐measured dose was then compared to the TPS‐calculated dose to quantify the accuracy of TPS calculations at various distances from the field edge and out‐of‐field anatomical locations of interest (i.e., radiosensitive organs). The individual components of out‐of‐field dose (patient scatter, collimator scatter, and head leakage) were also calculated in Pinnacle and compared to Monte Carlo simulations for a $10\times 10\;{\text{cm}}^{2}$ field. Our results show that the treatment planning system generally underestimated the out‐of‐field dose and that this underestimation worsened (accuracy decreased) for increasing distances from the field edge. For the three IMRT treatment plans investigated, the TPS underestimated the dose by an average of 50%. Our results also showed that collimator scatter was underestimated by the TPS near the treatment field, while all components of out‐of‐field dose were severely underestimated at greater distances from the field edge. This study highlights the limitations of commercial treatment planning systems in calculating out‐of‐field dose and provides data about the level of accuracy, or rather inaccuracy, that can be expected for modern IMRT treatments. Based on our results, use of the TPS‐reported dose could lead to an underestimation of secondary cancer induction risk, as well as poor clinical decision‐making for pregnant patients or patients with implantable cardiac pacemakers and defibrillators.

PACS numbers: 87.53.Bn; 7.55.D‐

## I. INTRODUCTION

During external‐beam radiation therapy, the patient receives dose outside of the primary radiation field due to secondary radiation sources, including photon leakage through the treatment head of the accelerator (“head leakage”), scattered radiation from collimators and beam modifiers (“collimator scatter”), and internal patient scatter. Kase et al.^(^ ^1^ ^)^ measured these components of out‐of‐field dose separately and found that patient scatter is the main dose contributor near the field edge, while leakage radiation becomes the major contributor at large distances from the field edge. A motivating factor for understanding the individual components of out‐of field dose and how they can be reduced is the reduction in the risk of developing a secondary primary malignancy (SPM) following radiation therapy, which has become a major concern in the past decade. It has been found that the cumulative incidence of SPM could be as high as 20% of patients treated with radiation therapy. The incidence of these secondary malignancies depends on the delivered dose distribution, size of the irradiated volume, dose, and dose rate, along with other patient‐specific factors.^(^ ^2^ ^)^ Diallo et al.^(^ ^3^ ^)^ found that the majority of these second cancers arise in the margin of the irradiated region or the “beam‐bordering” region (from 2.5 cm inside to 5 cm outside of the irradiated volume) and that a sizeable number of cancers developed at distant sites far outside of the treatment field. In order to estimate the risk of developing a secondary malignancy, as well as to better understand the dose‐carcinogenic effect relationship, accurate knowledge of the dose distribution delivered to the patient is required, especially in the beam‐bordering region and out‐of‐field region. Aside from research regarding secondary cancers, accurate knowledge of this peripheral dose is also important in many clinical situations, such as the treatment of pregnant patients or patients with implanted electronic devices like pacemakers. In these cases, clinical decision‐making is based on dose thresholds (e.g., 2 Gy for implanted cardiac pacemakers), and thus inaccurate dose knowledge could lead to poor decision‐making.^(^ ^4^ ^)^

Since intensity‐modulated radiation therapy (IMRT) treatments are associated with a greater number of monitor units and thus greater levels of out‐of‐field dose in comparison to conventional radiation therapy, this treatment modality is especially of interest when discussing out‐of‐field dose and secondary cancer risk.^(^ ^5^ ^)^ This increased dose is particularly of concern for pediatric patients because children have a greater risk of developing a secondary cancer due to their tissue's higher radiation sensitivity and their longer survival times.^(^ ^6^ ^,^ ^7^ ^)^

Radiation therapy treatment planning systems (TPSs) are not commissioned for out‐of‐field dose calculations,^(^ ^8^ ^,^ ^9^ ^)^ and it is generally accepted that the accuracy of out‐of‐field dose calculations by TPSs is poor. Howell et al.^(^ ^10^ ^)^ quantified the accuracy of out‐of‐field dose calculations by the analytic anisotropic algorithm (AAA) of the Eclipse TPS for a simple mantle field and found that the TPS underestimated out‐of‐field doses by an average of 40% over the range of locations investigated. On the other end of the treatment complexity spectrum, Court et al.^(^ ^11^ ^)^ and Jang et al.^(^ ^12^ ^)^ evaluated the dose calculation accuracy of commercial TPSs for IMRT mesothelioma treatments.

Although the out‐of‐field dose calculation accuracy of commercial treatment planning systems has been shown in certain cases to be poor, general evaluations of this inaccuracy have received little attention. Consequently, it is not generally known under what circumstances TPS‐calculated doses are no longer reasonably accurate, which impedes both clinical and research decisions. Because the accuracy of out‐of‐field doses calculated by commercial TPSs varies with the distance from the field edge and the depth in the patient,^(^ ^10^ ^)^ it would be valuable to investigate calculation accuracy for a variety of out‐of‐field locations and radiosensitive organs for clinically relevant IMRT treatment plans. Therefore, the objective of this study was to quantify the accuracy of out‐of‐field dose calculations by the ${\text{Pinnacle}}^{3}$ v.9.0 TPS (Philips Healthcare, Andover, MA) for three clinically relevant IMRT cases: a pediatric brain case, a lung case, and a breast case. Furthermore, the accuracy of each out‐of‐field dose component (head leakage, collimator scatter, and patient scatter) was evaluated in order to pinpoint weaknesses in the TPS that lead to poor accuracy in out‐of‐field dose calculations.

## II. MATERIALS AND METHODS

### A. Treatment planning

**Figure 1:**
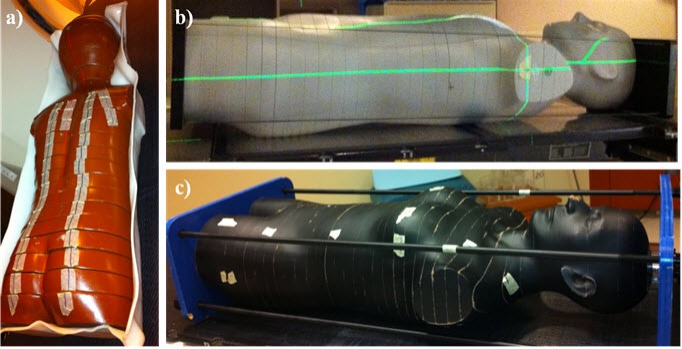
Photographs of the (a) pediatric anthropomorphic phantom used for the pediatric brain treatment plan, (b) adult reference male anthropomorphic phantom used for the thoracic treatment plan, and (c) adult female anthropomorphic phantom used for the breast treatment plan.

Three different anthropomorphic phantoms were used in this study (Fig. 1), one for each of the three IMRT treatment plans (for lung, breast, and pediatric brain cancers). For each phantom, a clinically relevant 6 MV step‐and‐shoot IMRT treatment plan was created in the ${\text{Pinnacle}}^{3}$ v.9.0 TPS using treatment planning guidelines from our institution, the specifics of which are described below for each case. Treatment plans were optimized using Pinnacle's inverse planning Direct Machine Parameter Optimization (DMPO) algorithm to achieve acceptable target coverage while minimizing dose to local avoidance structures. All dose distributions were calculated using the collapsed cone convolution superposition algorithm and a dose grid of $4\times 4\times 4\;{\text{mm}}^{3}$. Furthermore, dose distributions for each of the three IMRT treatment plans were recalculated using a $2\times 2\times 2\;{\text{mm}}^{3}$ grid size to investigate any changes in the accuracy as compared to the coarser $4\times 4\times 4\;{\text{mm}}^{3}$ grid size. The Pinnacle TPS does model several out‐of‐field parameters including effective source size, which models the penumbra of the beam by blurring the incident fluence with a Gaussian blurring kernel, and parameters that model scatter originating from the flattening filter. Although leakage radiation is not explicitly modeled as its own parameter, MLC and jaw transmission are included in the beam model.^(^ ^9^ ^,^ ^13^ ^)^

#### A.1 Pediatric brain case

For the pediatric brain cancer case, an anthropomorphic phantom representing a 5‐year‐old was used for treatment planning and measurements (Alderson Radiation Therapy Phantom, Radiology Support Devices, Long Beach, CA). A treatment plan was created with five noncoplanar beams and a prescribed dose of 60 Gy (2.4 Gy/fraction, 25 fractions) to a hypothetical planning target volume (PTV). The target volume was $39{\text{cm}}^{3}$ and was located in the brainstem. Avoidance structures used in the plan optimization process included the left and right eye and lens, optic chiasm, brainstem, and local nontarget brain tissue. The final plan required 186 MUs per gray of prescribed dose.

#### A.2 Lung case

An ATOM anthropomorphic male reference phantom (CIRS, Inc., Norfolk, VA) was used for the lung cancer treatment plan and included tissue‐equivalent materials for lung, bone, brain, and soft tissue.^(^ ^14^ ^)^ A seven coplanar beam IMRT treatment plan was created with a prescription dose of 66 Gy (2 Gy/fraction, 33 fractions) to a hypothetical PTV ($793{\text{cm}}^{3}$ volume) located in the medial superior lobe of the left lung. The total lung, spinal cord, heart, and local nontarget tissue were used as avoidance structures in the plan optimization process. The final plan required 242 MUs per gray of prescribed dose.

#### A.3 Breast case

A custom‐built adult female anthropomorphic phantom designed with realistic breast size and shape (RANDO, The Phantom Laboratory, Salem, NY) was used for the breast cancer treatment plan. A field‐in‐field treatment plan with four beams (two lateral and two medial fields) was created with a prescription dose of 50 Gy (2 Gy/fraction, 25 fractions) to the isocenter and covering the entire left breast, requiring 121 MUs per gray of prescribed dose.

### B. Phantom irradiation

All three treatment plans were delivered using 6 MV photons and a Clinac 2100 linear accelerator equipped with a 120‐leaf MLC (Varian Medical Systems, Palo Alto, CA). Dose measurements were performed with LiF TLD‐100 powder capsules, which are cylindrical tubes with approximate dimensions of 0.5 cm height and 2 mm diameter (Harshaw Chemical Company, Solon, OH). These detectors have been shown to be accurate out‐of‐field dosimeters for linear accelerators operated at 6 MV,^(^ ^15^ ^)^ and TLD measurements in an anthropomorphic phantom are widely used for determining peripheral organ doses for photon radiation therapy.^(^ ^10^ ^)^ For each of the three treatment plans, measurement points were chosen to obtain information at a variety of distances away from the field edge, depths, and radiosensitive organs of interest. In general, TLDs were not placed near the surface of the phantom or at lung/tissue interfaces due to the known limitations of the collapsed cone convolution superposition algorithm at material interfaces.^(^ ^16^ ^)^ However, for certain anatomical sites (e.g., the superficial breast buds), this was unavoidable. The number of measurement locations for each treatment plan ranged from 23 for the pediatric brain case to 29 for the lung case. TLDs were placed in a variety of out‐of‐field locations, with the distance between the measurement point and the field edge ranging from less than 1 cm to more than 30 cm, including several in‐field, low‐dose locations. Anatomical points of interest chosen for TLD measurement were out‐of‐field radiosensitive organs that are at risk for developing secondary malignant neoplasms or other deleterious radiation effects; the specific radiosensitive organs chosen for each treatment plan are listed in Table 1. Several fractions of each treatment were delivered to ensure that the dose delivered to each TLD fell between 5 cGy and 6 Gy. For each measurement point, two TLD capsules were inserted into the predrilled holes in the phantom slabs, yielding two readings which were averaged to obtain the TLD‐measured dose for that measurement location.

**Table 1:** The mean TLD‐measured dose ($D_{\text{meas}}$) and mean TPS‐calculated dose ($D_{\text{calc}}$) for various organs of interest associated with each of the three IMRT cases.

*Case*	*Organ of Interest*	*Mean Distance from Field Edge (cm)* ^b^	*Mean* $D_{meas}\left(cGy/Gy_{Rx}\right)$	*Mean* $D_{calc}\left(cGy/Gy_{Rx}\right)$	$D_{meas}/D_{calc}$
	Spinal cord (1)^a^	1.6	10.52	12.00	1.14
	Eye lens (2)	3.6	10.59	7.61	0.68
	Thyroid (2)	6.9	0.88	0.58	0.67
	Breast buds (2)	15.5	0.29	0.19	0.65
Pediatric Brain	Lungs (4)	18.3	0.27	0.14	0.48
	Heart (5)	20.1	0.20	0.01	0.05
	Ovaries (1)	39.0	0.06	0	0
	Heart (3)	2.3	30.67	31.09	1.01
Lunt	Contralateral lung (8)	6.3	5.87	5.12	0.86
	Thyroid (2)	7.3	1.56	0.91	0.59
	Ovaries (2)	33.8	0.12	0.03	0.27
	Contralateral breast (2)	3.8	2.85	2.46	0.80
	Heart (2)	4.6	2.79	1.98	0.70
Breast	Ipsilateral lung (3)	6.1	2.35	1.42	0.58
	Thyroid (2)	7.4	0.66	0.69	1.04
	Contralateral lung (2)	13.2	0.94	0.48	0.52
	Ovaries (2)	39.1	0.07	0.01	0.10

a The number of measurement points for each specific organ at risk is denoted in parenthesis.

b The mean distance from the field edge was measured from the 50% isodose line.

### C. TLD analysis

TLD readout and analysis was performed at the Radiological Physics Center (Houston, TX), which has a well‐established protocol for TLD analysis.^(^ ^17^ ^,^ ^18^ ^)^ For this study, standards were irradiated with a Co‐60 unit in order to obtain the system calibration factor (*C*_*D,w*_). In addition to the standard energy response correction factor that accounts for the difference in TLD response to a 6 MV spectrum relative to Co‐60 photons ($k_{E}$), an additional energy correction factor was used to account for the softer beam spectra observed outside of the treatment field ($k_{NR}$); these nonreference (out‐of‐field) energy correction factors were taken from Scarboro et al.^(^ ^19^ ^)^ Thus, the TLD absorbed dose was determined by Eq. (1), where *D* is the absorbed dose, *M* is the raw TLD signal per unit mass of TLD powder, $k_{L}$ is the linearity correction factor, and $k_{F}$ is the fading correction factor:

(1) $$D=M\times C_{\text{D,W}}\times k_{L}\times k_{F}\times k_{E}\times k_{\text{NR}}$$

The uncertainty of the dose calculation (including the added uncertainty of the nonreference energy correction factor) for each TLD reading is ≤4%.^(^ ^18^ ^)^

TPS‐reported doses were determined by contouring a region of interest (ROI) approximately the shape and dimensions of a TLD capsule and taking the mean ROI dose. The percent error between the TLD‐measured dose and TPS‐calculated dose for all measurement points was calculated as a function of distance from the field edge, taking the TLD‐measured dose as the true dose and the distance from the field edge as the distance from the measurement location to the 50% isodose line.

### D. Accuracy of individual out‐of‐field dose components for open field

In order to gain a better understanding of the source of TPS calculation inaccuracies, the accuracy of each individual component of peripheral dose was evaluated, paralleling the methodology described by Kase et al.^(^ ^1^ ^)^ and Kry et al.^(^ ^20^ ^)^ The total dose outside of the treatment field *T* was thereby decomposed into leakage radiation *L*, scatter from collimators and other beam line components *C*, and patient scatter *P*:

(2) T=L+C+P

Using a large acrylic phantom and a $10\times 10\;{\text{cm}}^{2}$ field, the total out‐of‐field dose T was calculated in Pinnacle at various distances from the field edge. The combination (L+C) of head‐leakage and collimator‐scatter was calculated by directing the beam just outside of the phantom, thereby eliminating the patient scatter component (*P*). The leakage radiation component (*L*) was calculated by directing the beam outside of the phantom, closing the MLCs completely, and adding a lead block beneath the collimators, thereby eliminating patient scatter and collimator scatter. Thus, the individual components of out‐of‐field dose (*L*, *C*, and *P*) could be calculated based on knowledge of *T*, *L*, and (L+C). These results from Pinnacle were then compared to previously published Monte Carlo results simulated with a detailed model of a Varian 2100 Clinac, the same experimental setup conditions, and a parallel computational approach.^(^ ^20^ ^)^

## III. RESULTS

### A. Out‐of‐field dose accuracy for IMRT treatment plans

**Figure 2:**
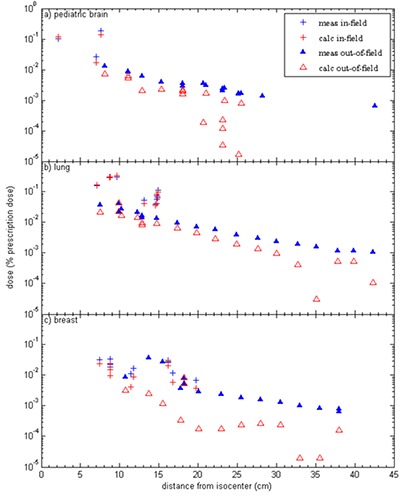
The TLD‐measured and TPS‐calculated dose, expressed as a percentage of the prescription dose, plotted as a function of distance from isocenter for: (a) pediatric brain, (b) lung, and (c) breast IMRT treatment plans. (▴) indicates out‐of‐field doses; (+) indicates in‐field, low doses.

In Fig. 2, the TLD‐measured doses and the TPS‐calculated doses are plotted as a function of distance from the treatment isocenter for each of the three IMRT treatment plans. This figure includes measurement points outside the treatment field (denoted with triangles) as well as low‐dose points inside the treatment field (denoted with crosses). For all three plans, the treatment planning system generally underestimated the dose in comparison to the measured dose. This was true for both low‐dose in‐field locations, as well as out‐of‐field locations, but the magnitude of underestimation was larger for out‐of‐field locations. The TLD‐measured dose fell off approximately exponentially as the distance from the isocenter increased; this result is similar to conventional field data from the Report of American Association of Physicists in Medicine Task Group 36.^(^ ^21^ ^)^ Although the TPS‐calculated doses follow a similar trend for higher doses, the calculated doses do not demonstrate this exponential falloff behavior for locations far outside the treatment field; rather, they exhibited less predictable, almost random, behavior.

**Figure 3:**
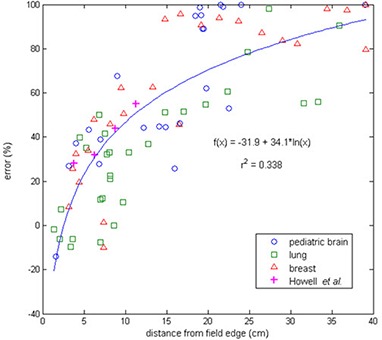
The error (%) between TPS‐calculated dose and TLD‐measured dose plotted as a function of distance from the field edge (the 50% isodose line) for the pediatric brain, lung, and breast IMRT treatment plans. Data from the study by Howell et al.^(^ ^10^ ^)^ with a simple mantle field and the Eclipse TPS is included for comparison.

Figure 3 shows the percent error between the TLD‐measured dose and TPS‐calculated dose for all measurement points (both out‐of‐field locations and low‐dose in‐field locations) in all three IMRT treatment cases, plotted as a function of distance from the field edge. The mean error was 61% for the pediatric brain plan, 33% for the lung plan, and 61% for the breast plan. For all three plans, the percent error was near zero at the field edge and increased as the distance from the field edge increased, approaching 100% at large distances for which the treatment planning system reported zero dose. Of note, dose calculation errors in excess of 30% were found as little as 5 cm from the field edge.

For the data displayed in Figs. 2 and 3, the TPS‐calculated dose was calculated with a $4\times 4\times 4\;{\text{mm}}^{3}$ grid size in Pinnacle. The dose distributions were also calculated with a finer dose grid resolution ($2\times 2\times 2\;{\text{mm}}^{3}$). However, the finer resolution did not affect the TPS‐calculated dose substantially, and the overall impact on the error between TLD‐measured and TPS‐calculated dose was <1% for the majority of measurement points.

Table 1 lists the ratio of TPS‐calculated dose to TLD‐measured dose $D_{\text{calc}}/D_{\text{means}}$ for the specific out‐of‐field organs of interest for each of the IMRT cases; $D_{\text{calc}}/D_{\text{means}}$ values ranged from 0.00 (100% underestimation of dose) to 1.14 (14% overestimation of dose). Once again, it is clear that the accuracy of the TPS generally worsened, exemplified by $D_{\text{calc}}/D_{\text{means}}$ deviating more from unity, as the distance of the organ of interest from the field edge increased. However, substantial underestimation of dose by the TPS occurred even for organs located close to the treatment field. For instance, the contralateral breast (mean distance of 3.8 cm from field edge) and ipsilateral lung (mean distance of 6.1 cm from the field edge) doses from the breast treatment plan were underestimated by 20% and 42%, respectively.

### B. Accuracy of out‐of‐field dose components for open field

**Figure 4:**
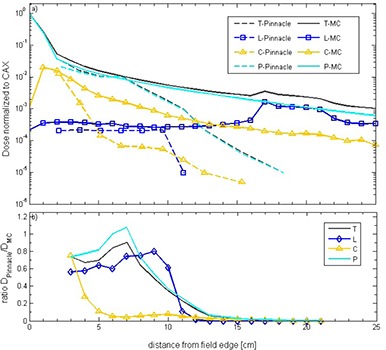
Out‐of‐field dose components (T= total, L= head leakage, C= collimator scatter, and P= patient scatter) calculated by the Pinnacle TPS and Monte Carlo simulations (MC) plotted as a function of distance from the field edge (a), along with ratio of the two (b), for a $10\times 10\;{\text{cm}}^{2}$ open field.

For a simple $10\times 10\;{\text{cm}}^{2}$ open field, Fig. 4(a) shows the TPS‐calculated dose, as well as the Monte Carlo dose, plotted as a function of distance from the field edge for the total out‐of‐field dose, as well as each of the three individual components. The ratio of the TPS‐calculated dose to the Monte Carlo dose is shown in Fig. 4(b). Near the edge of the treatment field (within 10 cm of the field edge), the TPS severely underestimates the contribution from collimator scatter. Head leakage was consistently underestimated (by approximately 30%) up to 10 cm from the field edge, but beyond this distance the TPS‐calculated head leakage dose dropped to approximately zero. Because head leakage is the dominant source of out‐of‐field dose at large distances from the field edge,^(^ ^1^ ^)^ this lack of head leakage modeling by the TPS is the dominant source of error beyond approximately 12 cm from the field edge. Patient scatter was the best modeled component of out‐of‐field dose, being within 25% of the true value out to approximately 7 cm from the field edge. However, at larger distances, the error in this component increased substantially and rapidly.

## IV. DISCUSSION

For the three IMRT treatment plans in this study, the treatment planning system underestimated the out‐of‐field dose for almost all our measurements points, with this underestimation worsening as distance from the field edge increased. On average, the TPS‐calculated dose was 50% less than the measured value. While our TLD measurements showed an exponential dose falloff for increasing distances out‐of‐field, the TPS‐calculated doses did not follow this exponential behavior and appeared rather erratic for large distances out‐of‐field (Fig. 2). Although it has been shown in several studies that there is very little depth‐dependence for out‐of‐field dose measurements taken at a constant distance from the field edge,^(^ ^22^ ^,^ ^23^ ^)^ TPS‐calculated out‐of‐field doses do show depth dependence.^(^ ^10^ ^)^ Since measurement and calculation locations were not taken at a uniform depth in the anthropomorphic phantoms, this nonexponential behavior in the TPS‐calculated doses is likely attributable to this depth‐dependence.

Although the majority of our measurement data showed that the TPS underestimated the dose, there were a several measurement locations at which the TPS overestimated the dose. These points were close to the field edge (usually within 5 cm) and thus experienced the largest dose gradients (Fig. 3). Therefore, we believe that small positional errors in the TLD measurements are the cause of this effect rather than TPS inaccuracy near the target volume. We crudely estimated the magnitude of the dose error associated with positioning uncertainty in high gradient regions by taking the standard deviation of the TPS‐calculated dose over the ROI corresponding to the TLD. This value was typically 5%–15% for our measurement points, while the uncertainty associated with individual TLD readings was ≤4%.^(^ ^18^ ^)^ This gives a rough estimate of overall uncertainty of 6%–16%, for a conservative estimate of positional uncertainties for measurements in high dose gradient regions. In general, the error associated with TPS dose calculation inaccuracies (Fig. 3) was much greater than the uncertainty associated with our TLD measurements for nearly all of our data points. Therefore, if measurements in high dose gradients cannot be avoided, it would still be worthwhile to perform these measurements, despite the high uncertainties associated with positioning errors, because the results would still be more accurate than simply using the TPS‐calculated dose.

The data in the current study were compared to previous findings in Fig. 3, which also shows data from the study by Howell et al.^(^ ^10^ ^)^ investigating out‐of‐field dose for the Eclipse treatment planning system. It is evident that the data from our study agree well with those of Howell and colleagues, although there is greater spread in the data from the current study. This is particularly interesting for two reasons. First, the two studies used different treatment planning systems. Second, while the Howell study considered conventional therapy, the current work used IMRT. The general agreement between the two datasets suggests that, at least for the cases in this study, IMRT does not substantially increase the inaccuracy of out‐of‐field dose calculations by commercial treatment planning systems. This conclusion is further supported by the general agreement between the three IMRT cases in the current study, despite the differences in the degree of modulation. The breast plan was the least modulated, while the thoracic plan was the most modulated. However, if anything, better agreement was observed for the thoracic case (Fig. 3).

In order to pinpoint weaknesses within the treatment planning system that cause poor accuracy out‐of‐field, we quantified the error in dose calculation associated with each individual component of dose. Although this data was obtained for a simple $10\times 10\;{\text{cm}}^{2}$ open field rather than a complex IMRT treatment plan, it is nonetheless valuable for understanding the fundamental reasons why the TPS cannot accurately calculate out‐of‐field dose. Not only does the TPS severely underestimate collimator scatter and scatter from other beam line components, but it also underestimates patient scatter. The severity of these underestimations increased for increasing distances from the field edge. Furthermore, the leakage radiation from the accelerator head is underestimated, especially at distances greater than 10 cm from the field edge, where Pinnacle appears to stop modeling leakage radiation and thus reports zero dose due to this component. Of note, this poor accuracy is not due to poor beam modeling in the TPS but rather more fundamental sources. Even if the collimator scatter and leakage radiation were better modeled in the TPS, the patient scatter component, which is the dominant component near the field, will still be underestimated due to the underestimation of large angle scatter, a shortcoming of commercial implementations of the convolution/superposition dose calculation method. We verified that adjusting the beam model would not meaningfully improve the accuracy of dose calculations out‐of‐field by recalculating our measurement points using an alternative clinical beam model that exhibits sharper beam penumbra and increased jaw and MLC transmission. This alternate beam model did not result in a change in overall accuracy. An interesting implication of these TPS inadequacies is that despite the different out‐of‐field dose distributions associated with new treatment techniques, such as volumetric‐modulated arc therapy (VMAT) and radiation therapy with flattening filter‐free (FFF) beams, the out‐of‐field accuracy of the TPS should not be expected to be different from that of IMRT because the same fundamental limitations in the TPS exist. However, the current study was limited to step‐and‐shoot IMRT only, and future studies are needed to quantify the accuracy for these new treatment techniques.

A limitation of this study is that only 6 MV photons were investigated. It would be also be interesting to quantify the error associated with TPS‐calculated out‐of‐field doses for IMRT treatments delivered at higher photon energies (e.g., 10 MV and 18 MV). Higher energy treatments should be associated with more leakage radiation and less patient scatter. Based on Fig. 4, the TPS models patient scatter better than the other components of out‐of‐field dose, suggesting that the accuracy could be worse for higher energy photon treatments. Furthermore, since neutrons are produced at energies above 10 MV and neutron dose is not accounted for in the TPS, neutron dose could be another source of error that worsens out‐of‐field TPS accuracy at higher photon energies. Further studies are needed to investigate the accuracy for higher energy photons, which is beyond the scope of the current study.

These findings are relevant to many aspects of clinical care and radiotherapy research. For pregnant patients or patients with implanted electronic devices, it is important to accurately assess the dose to the fetus or device to guide clinical management. The current work found that even at 3–4 cm from the field edge, the TPS could underestimate the dose by 30% or more. Dose assessment at such locations should therefore not generally rely on TPS calculations. Similarly, for research or clinical investigations into the potential for second cancer development, the TPS should generally not be used to assess the risk associated with out‐of‐field locations. At low to intermediate doses, the risk of a second cancer increases as the dose increases; this relationship is linear for low doses.^(^ ^24^ ^)^ Therefore, for organs receiving low doses, underestimating the dose received by a radiosensitive organ by 50% by using the TPS‐calculated dose leads to a 50% underestimation in risk.

## V. CONCLUSIONS

In this study, we quantified the accuracy of out‐of‐field dose calculations by the Pinnacle treatment planning system for three IMRT treatment plans and found that the treatment planning system underestimated the out‐of‐field dose by an average of 50% at our measurement locations, with the degree of dose underestimation increasing with greater distance from the field edge. Locations relatively close to the treatment field (within 3–4 cm) could be associated with TPS‐calculation errors in excess of 30%, while far from the field edge the error approaches 100%. Because a severe underestimation of out‐of‐field dose to an organ can lead to a severe underestimation of the risk of developing a secondary malignancy, as well as poor clinical decision‐making for pregnant patients and patients with implantable electronic devices, TPS‐reported peripheral doses should generally not be used in these cases. The source of these TPS errors appears to be underestimation of scattered radiation from collimators and other beam modifiers in the near field, as well as underestimation of leakage radiation and internal patient scatter at greater distances from the field edge.

## ACKNOWLEDGMENTS

This work was supported by Public Health Service grants CA 10953 and CA 081647, awarded by the National Cancer Institute, Department of Health and Human Services. One of the authors, Jessie Huang, would like to acknowledge financial support from the Graduate School of Biological Sciences, UT Health Science Center at Houston. We would like to thank Zachary Bohannan and Kathryn Carnes for their assistance in editing this manuscript.
